# What will it take to cure hepatitis B?

**DOI:** 10.1097/HC9.0000000000000084

**Published:** 2023-03-24

**Authors:** Wen-Juei Jeng, Anna S.F. Lok

**Affiliations:** 1 Department of Gastroenterology and Hepatology, Chang Gung Memorial Hospital, Linkou Medical Center, Taoyuan, Taiwan; 2 College of Medicine, Chang Gung University, Taoyuan, Taiwan; 3 Division of Gastroenterology and Hepatology, University of Michigan, Ann Arbor, Michigan, USA

## Abstract

The current treatment of chronic HBV infection, pegylated interferon-α (pegIFNα) and nucleos(t)ide analog (NA), can suppress HBV replication, reverse liver inflammation and fibrosis and reduce the risks of cirrhosis, HCC, and HBV-related deaths, but relapse is common when the treatment is stopped before HBsAg loss. There have been major efforts to develop a cure for HBV, defined as sustained HBsAg loss after a finite course of therapy. This requires the suppression of HBV replication and viral protein production and the restoration of immune response to HBV. Direct-acting antivirals targeting virus entry, capsid assembly, viral protein production and secretion are in clinical trials. Immune modulatory therapies to stimulate adaptive or innate immunity and/or to remove immune blockade are being tested. NAs are included in most and pegIFNα in some regimens. Despite the combination of 2 or more therapies, HBsAg loss remains rare in part because HbsAg can be derived not only from the covalently closed circular DNA but also from the integrated HBV DNA. Achievement of a functional HBV cure will require therapies to eliminate or silence covalently closed circular DNA and integrated HBV DNA. In addition, assays to differentiate the source of circulating HBsAg and to determine HBV immune recovery, as well as standardization and improvement of assays for HBV RNA and hepatitis B core-related antigen, surrogate markers for covalently closed circular DNA transcription, are needed to accurately assess response and to target treatments according to patient/disease characteristics. Platform trials will allow the comparison of multiple combinations and channel patients with different characteristics to the treatment that is most likely to succeed. Safety is paramount, given the excellent safety profile of NA therapy.

## Graphical abstract

**Figure:**
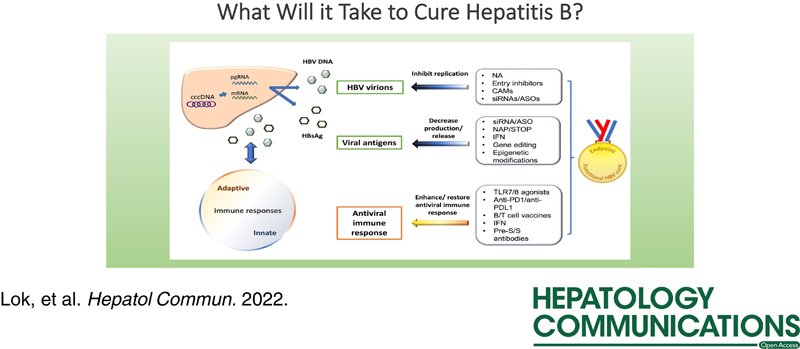

## INTRODUCTION

Hepatitis B virus (HBV) infection remains a global health problem despite the availability of safe and effective vaccines for 4 decades, with an estimated 1.5 million new infections, 296 million chronically infected persons, and 820,000 HBV-related deaths in 2019.^1^ Current treatment with pegylated interferon alfa (pegIFNα) or nucleos(t)ide analogs (NAs) can suppress HBV DNA replication and decrease liver inflammation.^2^ They have also been shown to reverse liver fibrosis and decrease the risk of cirrhosis, HCC, and liver-related deaths. However, HBsAg loss rarely occurs, and viral relapse is common when the treatment is stopped. Thus, most patients require long durations or lifelong treatment prompting major efforts to develop a cure for chronic hepatitis B. Many review articles summarizing the efficacy of new HBV direct-acting antiviral and immunomodulatory therapies in clinical trials have been published in recent years.^3^–^5^ This review article will focus on what is feasible in the foreseeable future and discuss what will be needed to achieve HBV cure.

## BARRIERS TO HBV CURE

**FIGURE 1:**
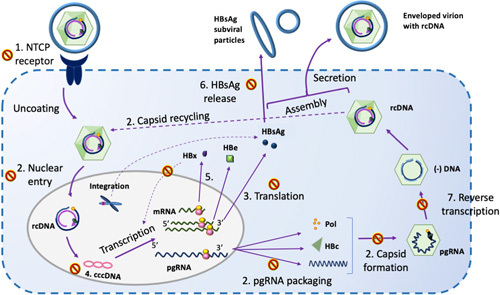
HBV life cycle and targets for direct-acting antiviral agents. Direct-acting antiviral drugs in development or clinical use include (1) Entry inhibitors targeting sodium taurocholate cotransporting polypeptide; (2) capsid assembly modulators (CAM) primarily interfering with capsid assembly but may also interfere with capsid disassembly and recycling; (3) translation inhibitors: small interfering RNA (siRNA), ASO, RNA destabilizers, locked nucleic acid (LNA)s that decrease viral protein and virion production; (4) cccDNA epigenetic modifiers, destabilizers, endonucleases; (5) HBx inhibitors that may decrease cccDNA transcription; (6) HBsAg release inhibitors such as nucleic acid polymers; (7) HBV DNA replication inhibitors such as nucleos(t)ide analogs (NAs), RNase H inhibitors. Numbers in the figure indicate the sites of action of each class of direct-acting antiviral agents. Abbreviations: cccDNA, covalently closed circular DNA; NTCP, sodium taurocholate cotransporting polypeptide; rcDNA, relaxed circular DNA.

The challenges to HBV cure relate to the reservoirs for HBV replication and antigen production covalently closed circular DNA (cccDNA) and integrated HBV DNA and the impaired innate and adaptive immune responses against HBV. The episomal cccDNA, located in the hepatocyte nucleus, serves as a transcriptional template for all HBV RNAs, including pregenomic RNA (pgRNA), which is reverse transcribed into HBV DNA and messenger RNAs, which are translated into viral proteins (Figure 1). cccDNA is derived not only from the incoming virions but also from the intracellular recycling of nucleocapsids.^6^ Elimination of cccDNA is believed to occur mainly through dilution at the time of cell division. The half-life of cccDNA in nondividing hepatocytes had been estimated to be years though a recent study suggested the half-life may be weeks or months.^6^–^8^ The dual source of cccDNA, its stability, the lack of direct effect of NA on its production, and the incomplete suppression of HBV DNA replication by NA explain why cccDNA concentrations and HBsAg levels are minimally decreased even after many years of undetectable circulating HBV DNA.^9^,^10^

The error in primer translocation during HBV DNA replication may result in double-stranded linear DNA instead of relaxed circular DNA.^11^ These double-stranded linear DNA may be integrated into host DNA. Recent deep-sequencing studies showed that the full-length S gene is often preserved in integrated HBV DNA and can be a source of HBsAg.^12^ A study into the differential effects of a small interfering RNA (siRNA) on HBsAg levels in HBeAg-positive versus HBeAg-negative patients revealed that integrated HBV DNA is the predominant source of HBsAg in HBeAg-negative chimpanzees and patients.^13^ CccDNA has long been held the Achilles heel of HBV cure; this new finding has led to the revelation that treatments aimed to achieve HBsAg loss will require not only targeting cccDNA but also integrated HBV DNA.^14^

Patients with chronic HBV infection have impaired innate and adaptive immune responses to HBV. Persistently high viral antigen levels had been incriminated to result in the exhaustion of HBV-specific T cells^15^ and possibly dendritic cells and natural killer (NK) cells.^16^,^17^ The recovery of HBV-specific immune responses had been demonstrated in patients with spontaneous, IFNα-induced or NA-induced HBeAg or HBsAg loss.^18^–^20^ A recent study showed that HBV-specific T-cell responses were enhanced in some patients who experienced a marked decrease in HBsAg levels during siRNA therapy.^21^ These data suggest that sustained suppression of HBV DNA replication and HBsAg production may restore HBV-specific immune responses in some patients, but immune modulatory therapies may still be needed in others.

## DEFINITION OF HBV CURE

At the 2016 American Association for the Study of Liver Diseases (AASLD) and the European Association for the Study of the Liver (EASL) HBV Treatment Endpoints Conference,^22^ HBV cure was categorized as sterilizing, functional, and partial. Sterilizing cure, defined as the elimination of cccDNA and integrated HBV DNA, was considered an ideal but unrealistic endpoint. Functional cure, defined as sustained (>6 months post-treatment) HBsAg loss with or without seroconversion to anti-HBs and undetectable HBeAg and serum HBV DNA after a finite course of therapy, was considered an achievable endpoint and the goal to strive for. Partial HBV cure, defined as HBsAg positive, HBeAg negative with undetectable serum HBV DNA after the discontinuation of a finite course of treatment, was considered an acceptable intermediate endpoint during the development of HBV cure.

The definition of HBV functional cure adopted in 2016 and remained in place at the 2019 EASL-AASLD HBV Treatment Endpoint Conference^23^ assumed that HBsAg loss reflects the complete suppression of cccDNA transcription. The recent finding that cccDNA is not the only source of HBsAg may explain why some new antivirals aimed to decrease cccDNA concentration or transcription have minimal impact on HBsAg levels. It also raises the question of whether the current definition of a functional HBV cure is an achievable endpoint. Indeed, at the 2022 AASLD-EASL HBV Treatment Endpoint Conference, it was debated whether HBsAg loss is a lofty goal and whether more realistic endpoints should be adopted as intermediates to encourage the continued development of new drugs for hepatitis B. Alternative endpoints proposed included the suppression of HBsAg to a very low level, complete suppression of cccDNA transcriptional activity as reflected by undetectable HBV RNA or hepatitis B core-related antigen (HBcrAg) using standardized, sensitive assays, or partial cure.

It should be noted that other reasons for choosing HBsAg loss as the anchor for functional HBV cure include the low risk of virologic relapse when treatment is stopped, given the desire for finite therapy, the potential for incremental clinical benefit compared with HBV DNA suppression without HBsAg loss,^24^ and removal of the stigma of HBV infection. Contrary to universal viral relapse when pegIFNα or NA is stopped before HBsAg loss, the discontinuation of pegIFNα or NA in patients who have confirmed HBsAg loss (2 negative HBsAg test results >6 mo apart) is rarely associated with viral relapse or seroreversion to HBsAg positive.^25^–^28^ Several studies have shown that HBsAg loss confers a further reduction in risk of HCC compared with HBV DNA suppression alone though there may be no incremental benefits on the risk of cirrhosis complications or liver-related mortality.^24^ Despite public education, many patients with chronic HBV infection have experienced discrimination, and becoming HBsAg negative would remove the stigma that haunts them.

Low end-of-treatment HBsAg level is the best predictor of HBsAg loss after NA withdrawal and a strong predictor of HBsAg loss in ongoing trials of new HBV therapies.^29^,^30^ This is not surprising, as HBsAg decrease is in the path of HBsAg loss; however, there are no data on the predictive value of subsequent HBsAg loss when treatment is discontinued after HBsAg level is reduced to very low levels such as <100 or <10 IU/mL. Data from the RETRACT-B study on NA withdrawal showed that lower levels are required for Asians than Whites to achieve HBsAg loss.^31^ In addition to race (likely a surrogate for the duration of infection or HBV genotype), the mode of action of the HBV drug may also determine the threshold level of HBsAg that predicts a high probability of HBsAg loss after treatment is stopped. Low HBsAg levels have also been shown to be associated with lower risks of HCC and hepatic decompensation among HBeAg-negative patients with low HBV DNA levels^32^ whether this observation in untreated patients in whom low HBsAg levels may reflect host immune control is also true for patients whose HBsAg levels were reduced by therapies targeting HBsAg production or secretion has not been determined.

The complete suppression of cccDNA transcriptional activity was the original premise of the 2016 definition of HBV functional cure because serum HBsAg levels correlate better with hepatic cccDNA concentrations than serum HBV DNA levels. In the last 5 years, many studies have shown that serum HBV RNA and HBcrAg levels are better surrogate markers for hepatic cccDNA transcriptional activity than serum HBsAg levels.^33^–^35^ Current assays for HBV RNA are not standardized and measure not only pgRNA but also messenger RNA and spliced RNA. The assays for HBcrAg measure a composite of viral antigens from the precore/core gene: HBcAg, HBeAg, and p22 core-related antigen. These assays are not standardized, and sensitivity is poor. The standardization of HBV RNA and HBcrAg assays, improvement in sensitivity and range of linearity, and specification of the proportions of individual components measured or development of assays for individual components, for example, pgRNA, would be needed before these markers can be accepted as measures of the complete suppression of cccDNA transcriptional activity in clinical trials (Table 1).

**TABLE 1:** HBV markers and their role in assessing HBV cure

HBV marker	Limits of quantification	Utility in clinical practice/trials	Limitations of marker/assay
HBV DNA	10–20 to 10^9^ IU/mL	Measure virion concentration Assess HBV replication	Does not reflect cccDNA concentration or transcription activity during NA treatment. HBV DNA not detected in current assays may be detected with more sensitive assays.
HBsAg	0.05 to 130–250 IU/mL before dilution; upper limits of quantification 52,000-125000 IU/mL after serial dilution^a^	Measure HBsAg in subviral particles and virions HBsAg loss is currently used to define functional cure	Cannot differentiate HBsAg from cccDNA versus integrated HBV DNA. Correlation with cccDNA transcriptional activity is low in HBeAg negative patients. Levels vary by HBV genotypes.
HBcrAg	10^3^ to 10^7^ U/mL^b^	Measure 3 proteins coded by precore/core region: HBcAg, HBeAg and 22kd precore protein Reflect cccDNA transcription activity	Correlate with cccDNA transcription activity. Current assays not standardized, limited sensitivity, unquantifiable levels in ~50% HBeAg negative patients.
HBV RNA	~10 to ~10^8^ U/mL^c^	Measure pregenomic RNA, messenger RNAs, and spliced RNAs Reflect cccDNA transcription activity Levels at the end of NA therapy predict the risk of relapse after NA withdrawal	Correlate with cccDNA transcription activity. Percentage of HBV RNA detected being pgRNA depends on the design of assay. Limited range of linearity, performance across HBV genotypes not verified.

^a^ Detection range for Abbott ARCHITECT: 0.05~250 IU/mL, suggested dilution is 1:500, not to exceed 1:999; Roche Elecsys HBsAg II quant II: 0.05~130 IU/mL, 20-52,000 IU/mL with 400-fold dilution; further manual dilution may be performed if result >52,000 IU/mL after 400-fold dilution.

^b^ Lower limit of detection is 2 log_10_ U/mL and linear range is validated for 3-7 log_10_ U/mL

^c^ Not standardized includes different RNA species in varying proportions, depending on the assay used.

Abbreviations: cccDNA, covalently closed circular DNA; HBcrAg, hepatitis B core-related antigen; NA, nucleos(t)ide analogs.

Partial cure is rarely achieved after the discontinuation of pegIFNα or NA therapy. In most patients who remain HBsAg positive, HBV DNA is detectable after stopping pegIFNα or NA though some patients may remain in clinical remission (HBV DNA <2000 IU/mL and alanine aminotransferase (ALT) <2 times the upper limit of normal). Whether it is possible to achieve partial cure with new therapies remains to be determined; however, caution must be exercised when treatment is stopped before HBsAg loss as severe flares, including hepatic decompensation, have been reported in clinical trials of new antiviral drugs.^36^,^37^

## CURRENT TREATMENTS RARELY LEAD TO HBsAg LOSS

### Interferon monotherapy

IFN exerts modest antiviral effects but it can enhance host innate and adaptive immune responses against HBV.^38^ An in vitro study suggested that IFN may have direct effects on the stability or transcription of cccDNA.^39^ This may account for the higher rates of HBsAg and HBeAg loss than NA therapy. After a 48-week course of pegIFNα treatment, 20%–25% of patients achieve a sustained decrease in HBV DNA levels, with HBsAg loss increasing from 2% to 3% at the end of treatment to 8%–14% after 3–5 years post-treatment follow-up.^40^,^41^ However, the high rate of HBsAg loss associated with pegIFNα therapy is mainly observed in genotype A,^42^ which is predominantly seen in the US and western Europe but not in endemic countries.

### NA monotherapy

NAs act primarily through inhibiting the reverse transcription of pgRNA to HBV DNA. Among the approved NAs, entecavir, tenofovir disoproxil fumarate (TDF), and tenofovir alafenamide are preferred because of their high antiviral potency and low risk of antiviral drug resistance. Despite DNA suppression, only 2%–5% of patients lost HBsAg after 10-year treatment.^43^ It has been estimated that it will take >40 years of continuous NA therapy for most patients to achieve HBsAg loss.^44^ Thus, NAs are usually administered for many years and often lifelong, particularly in patients with cirrhosis, to prevent severe hepatitis flares due to viral relapse.

Recent studies showed that although viral relapse is universal when NAs are stopped, HBeAg-negative patients who discontinued NA after at least 2–4 years of complete HBV DNA suppression had higher rates of HBsAg loss with a cumulative incidence of 13%–41% by post-treatment year 5–6 compared with <5% in patients who continued NA.^31^,^45^–^49^ It has been proposed that viral relapse may trigger HBsAg clearance by host immune response that has been restored after years of virus suppression. However, most studies found that post-withdrawal ALT flare does not predict HBsAg loss.^50^,^51^

Although the Asian Pacific Association for the Study of the Liver and the EASL guidelines recommend that NAs may be stopped in selected HBeAg-negative patients who have completed >2–3 years of treatment with undetectable HBV DNA and agree to close follow-up,^52^,^53^ this approach should be adopted with caution. Despite careful patient selection and exclusion of patients with cirrhosis pretreatment, rare cases of severe hepatitis flares and decompensations have been reported, and ~50% of patients had to resume treatment within 4 years of NA withdrawal.^31^,^47^,^54^ Two retrospective multicenter cohort studies, including patients from North America, Europe, and Asia, showed that Asians had a 6.8–8.3-fold lower odds of HBsAg loss compared with Whites who stopped NA.^31^,^54^ The RETRACT-B study cited above proposed an HBsAg cutoff <1000 IU/mL for Whites and <100 IU/mL for Asians for consideration of NA withdrawal.^31^ Using the data from that study, 46% Whites had end-of-treatment HBsAg <1000 IU/mL but only 15% Asians had end-of-treatment HBsAg <100 IU/mL; of these, 41% Whites and 33% Asians would be predicted to have HBsAg loss by year 4 post-treatment. This strategy needs to be validated in prospective studies, but existing data suggest that it may have minimal impact on HBsAg loss in Asian patients.

### Combination of IFN and NA

Most studies evaluating *de novo* combination of IFN and NA have not shown an improvement in HBsAg loss except for 1 study showing *de novo* combination of pegIFNα and TDF, resulted in a higher rate of HBsAg loss than pegIFNα or TDF monotherapy, but the benefit was mainly seen in patients with HBV genotype A.^55^ A meta-analysis showed that IFN add-on to NA or switching from NA to IFN may increase HBsAg loss,^56^ but IFN is associated with many side effects and the results in these highly selected patients may not be generalized.

## NEW THERAPIES AIMED TOWARD HBV CURE

**FIGURE 2:**
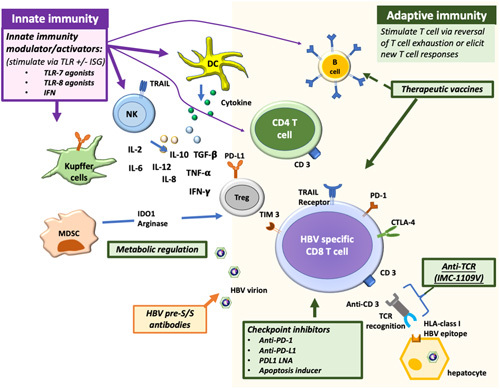
Immunomodulatory therapies aimed to restore innate and adaptive immune responses against HBV. Innate immunity modulators or activators include TLR7, 8 agonists, and IFN. Therapeutic vaccines, checkpoint inhibitors (anti-PD-1, Anti-PD-L1, PDL1 LNA, apoptosis inducer), and anti-HBV T-cell receptors (IMC-1109V) aim to restore or to stimulate the adaptive immune response to HBV by reversing T-cell exhaustion or triggering a new T-cell immune response. HBV pre-S/S antibodies aim to restore B-cell immune response by neutralizing circulating HBV virions/SVPs and may also prevent *de novo* infection of the hepatocyte. Abbreviations: IFN, interferon; MDSC, myeloid-derived suppressor cell; NK, natural killer; PD-1, programmed death receptor-1; TGF, transforming growth factor; TLR, toll-like receptor.

New drugs are needed to achieve functional HBV cure even if less ambitious endpoints are adopted; however, NAs will remain a backbone in suppressing HBV DNA replication, and pegIFNα may still play a role in decreasing HBsAg production or in facilitating HBsAg loss after it has been reduced to low levels. New therapies in development include direct-acting antivirals acting on different targets in the HBV life cycle, and immune modulators aimed to revive or stimulate HBV-specific immune response or to remove immune blockade (Figures 1 and 2). Most of these new drugs are tested in combination with NAs and some with pegIFNα. The results of some of the key trials are summarized below (Table 2, http://links.lww.com/HC9/A187).

**TABLE 2:** Summary of phase 2 clinical trial results of direct-acting antivirals and immunomodulatory therapies on HBV markers

	HBV DNA level (log_10_ IU/mL)	HBsAg level (log_10_ IU/mL)	HBsAg loss	HBcrAg level (log_10_ U/mL)	HBV RNA level (log_10_ U/mL)	Comments
Drug class: translation inhibitors						
siRNA						
JNJ-3989 VIR-2218 AB-729	1–2 log decline at 24 wk in absence of NA	1–3 log decline, plateau ~week 16 despite the continued dosing. >2 log decline at week 24 when combined with pegIFNα >12 wk	Rare, not sustained. 10 of 64 (15.6%) in VIR-2218 + pegIFNα arm lost HBsAg, 9 had anti-HBs at end of treatment, durability unknown.	~1 log decline at 24 wk, up to ~2 log decline in some NA-naive HBeAg+ patients	1–2 log decline at 24 wk, up to ~3 log decline in some NA-naive HBeAg+ patients	More marked HBV DNA suppression in combination with NA. HBsAg and HBV DNA remained suppressed at follow-up week 24 after 48 wk combination therapy with CAM and NA.
ASO						
GSK3389404 Bepirovirsen (GSK3228836) (B-clear)	2–2.6 log decline at 24 wk in absence of NA	1–3 log decline at 24 wk	26%–29% at the end of treatment in highest dose groups (300 mg × 24 wk), and 9%–10% (HBsAg <LLOD and HBV DNA <LLOQ) 24 wk post-treatment (0% in HBeAg+ patients not on NA), long-term durability unknown.	<1 log decline at 24 wk	1–2 log decline at 24 wk	More marked HBV DNA suppression in combination with NA. ALT flares (>3 times upper limit of normal) may occur with marked HBsAg decline.
Drug class: capsid assembly modulators (CAM)						
ABI-H0731 RG7907 (RO7049389) JNJ-6379 (JNJ-56136379) GLS4	1–4 log decline at 24 wk in absence of NA	<1 log decline at 24 wk	None	<1 log decline at 24 wk	>2 log decline at 48 wk	More marked HBV DNA and HBV RNA suppression in combination with NA. HBV DNA and pgRNA rebound post-treatment.
Drug class: HBsAg release inhibitor						
NAP						
REP-2139 REP-2165	Effect in absence of NA not available	1–6 log decline at 24 wk in combination with NA and pegIFNα	60% at end of 48 wk treatment in combination with NA and pegIFNα and 35% at 48 wk post-treatment follow-up	Not reported	Not reported	Effect on HBV DNA and HBsAg in absence of NA and pegIFNα not reported. Marked ALT flares common. Small number of patients, results remain to be confirmed.
Drug class: remove immune blockade						
Checkpoint inhibitor						
PDL1 inhibitor Envafolimab (ASC22)	Effect in absence of NA not available	<1 log decline at 24 wk	3 of 33 (9%) patients, all with baseline HBsAg <500 IU/mL	Not reported	Not reported	ALT flare observed in those with HBsAg reduction >0.5 log or HBsAg loss
Drug class: therapeutic vaccine						
GS-4774, yeast based, HBs, HBc, and HBx proteins	Effect in absence of NA not reported	GS-4774, <0.15 log decline at week 48 in combination with NA, 0.25 log decline at week 24 when combined with NA + Nivolumab.	1 of 13 (7.7%) GS-4774 + nivolumab lost HBsAg, durability unknown; none in GS-4774 alone arms	Not reported	Not reported	All 3 vaccines increase HBV-specific CD8 T-cell responses
VBI-2601, large, middle, and small HBs protein	—	<0.2 log decrease after 4 doses of VBI-2601.	None	—	—	—
VTP-300 (ChAdOx1/MVA), prime boost, inactivated polymerase, core, and S antigens		HBsAg <0.3 log decline 8 mo after last dose in most patients, more marked decline in combination with anti-PD-1	1 of 8 VTP-300 + Nivolumab lost HBsAg, durability unknown	—	—	—
Drug class: restore innate immunity						
TLR7 agonist						
GS-9620 (vesatolimod)	No additional decline when combined with NA	<0.5 log at 48 wk	None	Not reported	Not reported	ISG15 induction, increase T-cell and NK-cell response
TLR 8 agonist						
GS-9688 (selgantolimod)	No change	<1 log decline at 24 wk	2 of 33 NA+24 wk selgantolimod lost HBsAg at week 48, durability unknown	Not reported	Not reported	Induce cytokines that may activate antiviral effector function

Effects on HBV markers reported reflect that of new therapy in combination with nucleos(t)ide analog (NA) during treatment unless specified.

Abbreviations: HBc, hepatitis B core; HBs, hepatitis B surface; LLOD, lower limit of detection; LLOQ, lower limit of quantification; NA, nucleos(t)ide analogs; NAP, nucleic acid polymers; NK cell, natural killer cell; PD-1, programmed death-1; pegIFN, pegylated interferon-alpha; siRNA, small interfering RNA; TLR, toll-like receptor.

Please refer to Supplemental file for references. http://links.lww.com/HC9/A187

### Direct-acting antivirals

#### Entry inhibitors

Bulevirtide, a synthetic lipopeptide corresponding to the HBV preS1 region, blocks the entry of HBV into hepatocytes through the sodium taurocholate cotransporting polypeptide receptor, preventing infection of uninfected hepatocytes. Data on bulevirtide in chronic HBV monoinfection are limited. Studies in chronic HBV/HDV infection showed that bulevirtide alone had minimal effect on HBsAg decline with greater effect when used in combination with pegIFNα, but HBsAg loss is rare.^57^,^58^

#### Capsid assembly modulators

Capsid assembly modulators (CAMs) can interfere with multiple steps in the HBV life cycle. CAMs currently in clinical trials primarily act by interfering with capsid formation resulting in aberrant or empty capsids, thereby decreasing HBV DNA replication. New CAMs aim to achieve higher intracellular concentrations that are necessary for interference with capsid disassembly (cccDNA establishment) and with intracellular recycling of capsids (cccDNA replenishment).

Clinical trials of a combination of CAMs and NAs have shown an additive effect in decreasing HBV DNA and pgRNA levels but minimal effect on HBeAg and HBsAg levels suggesting that the main effect of CAM is interfering with pgRNA packaging and HBV DNA replication.^3^,^59^–^61^ The phase 2 trial of vebicorvir plus entecavir showed a more rapid and profound decline in HBV DNA and pgRNA levels compared with entecavir alone; however, there was a minimal decrease in HBsAg levels after 48-week treatment, and most patients who met the criteria for discontinuing treatment at week 72 had a viral relapse and many had hepatitis flares.^37^ Addition of vebicorvir to GalNAc-siRNA (AB-729) plus NA did not result in a greater on-treatment virological response compared with AB-729 and NA.^62^

#### Translation inhibitors

Translation inhibitors, including siRNAs and antisense oligonucleotide (ASO) ASO, silence HBV RNA, thereby inhibiting HBV DNA replication and viral protein production.

Clinical trials of the N-acetylgalactosamine-conjugated siRNAs (GalNAc-conjugated siRNAs) (JNJ-3989; VIR-2218; RG-6346; AB-729) showed 2–3 log_10_ HBsAg decline after 3–4 doses lasting for 6–9 months post-treatment and 38% had sustained >1 log_10_ HBsAg reduction 11 months from last dosing.^63^–^67^ In one trial of AB-729, HBsAg decline plateaued around 16–20 weeks despite the continued dosing to 48 weeks, and no patient achieved HBsAg loss.^68^ Mild ALT elevation and upregulation of HBV-specific T-cell activation markers in association with HBsAg decline was observed in some but not all patients.^21^ To date, clinical trials of siRNAs with up to 48 weeks of treatment with or without NA have not resulted in sustained HBsAg loss.^69^,^70^ In HBeAg-negative patients who achieved HBsAg <100 IU/mL with AB-729+NA and who discontinued treatment, sustained low HBV DNA and HBsAg level (−1.05 to −2.34 log_10_ from pretreatment level) without ALT flares was observed after up to 44 weeks follow-up.^71^ In a phase 2 trial combining 12–20 weeks of VIR-2218 (siRNA) with a neutralizing monoclonal antibody VIR-3434, most participants had absolute HBsAg level <10 IU/mL at the end of treatment, but none lost HBsAg.^72^ Another trial of VIR-2218 showed that the addition of peg-IFNα led to greater decrease in HBsAg level, with 10 of 64 (15.6%) patients who received combination therapy having HBsAg loss by the end of treatment and higher rates of response in those with longer (48 wk) duration of treatment.^73^ However, it is unclear whether HBsAg loss will be sustained off-treatment.

A phase 2 trial of bepirovirsen, an unconjugated ASO targeting all HBV RNAs, showed a marked decrease in HBsAg level at week 24 in the highest dose (300 mg) group, with 26% and 29% having undetectable HBsAg at the end of treatment, and 9% and 10% having undetectable HBsAg as well as unquantifiable HBV DNA after 24-week post-treatment follow-up, in patients with versus without concurrent NA, respectively.^29^ ALT flares (>3 times the upper limit of normal) were observed in patients with marked HBsAg decline,^29^ suggesting HBV immune reconstitution.^74^

The REEF-1 trial evaluated a triple combination of an siRNA (JNJ-3989), a CAM (JNJ-6379), and NA. Surprisingly, the triple combination resulted in less marked decrease in HBsAg level than the dual combination of siRNA and NA.^75^ Similar results were found in another study suggesting that CAM interference with siRNA may be a class effect.^62^

#### S secretion inhibitors

Nucleic acid polymers (NAPs) and S-antigen traffic inhibiting oligonucleotide polymers can block the release of subviral particles from hepatocytes.^76^,^77^ A study of 40 patients receiving TDF and pegIFNα with or without a NAP showed that 60% patients had HBsAg loss at the end-of-treatment and 35% had sustained HBsAg seroconversion >6 months after stopping treatment.^78^ This is the highest rate of functional HBV cure reported so far, but this trial involved a small number of patients, most patients had marked ALT flares (>1000 U/L in some patients), and the results of confirmatory studies have not been reported.

### Immune modulators

#### Innate immune response

Clinical trials of toll-like receptor 7 (TLR7) and TLR8 agonists showed minimal clinical efficacy despite increased cytokine production by T cells and NK cell activation.^79^–^81^

#### HBV-specific T-cell response

Previous attempts with therapeutic vaccines have not resulted in clinical efficacy.^82^ Two trials of GS-4774, a yeast-based vaccine, which expresses HBV S, X, and core antigens, in NA-treated virally suppressed and in NA-naive patients showed minimal reduction in serum HBsAg levels even in the presence of increased cytokine production by HBV-specific CD8 T cells.^83^,^84^ New approaches to therapeutic vaccines include the use of viral vectors, antigens from HBV core or polymerase regions, and combination with immune checkpoint inhibitors.^83^–^88^ A phase 1b/2a trial of Chimpanzee adenoviral and modified vaccinia Ankara viral vectors encoding multiple HBV antigens (ChAdOx1-HBV/MVA-HBV) used in a prime-boost strategy in virally suppressed patients showed that the addition of low-dose nivolumab (programmed death receptor-1 inhibitor) resulted in greater HBsAg decline (mean 1 log_10_ at 6 months) and 1 patient lost HBsAg lasting 8 months after the last dose.^89^

Other approaches, including autologous T-cell engineering with chimeric antigen receptor and T-cell receptor–redirected T cells, and soluble T-cell receptors linked to anti-CD3 antibodies (ImmTavs) are in preclinical or early phase clinical trials.^90^,^91^

#### HBV-specific B-cell response

Infusion of anti-HBs can neutralize circulating HBsAg and prevent the new infection of hepatocytes, but effects would be short lived unless HBsAg production is decreased and delivery of anti-HBs sustained. Several strategies to provide sustained delivery of anti-HBs, including the use of viral vectors, are being explored. Adding 12 weeks of a neutralizing monoclonal antibody VIR-3434 to VIR-2218 (siRNA) decreased HBsAg level to <10 IU/mL at the end of treatment in most patients though none achieved HBsAg loss.^72^

#### Removing inhibitory blockade

Checkpoint inhibitors such as programmed death receptor-1 blocking antibodies remove the blockade on T-cell function. A phase 1b study of 22 patients receiving 12 weeks of nivolumab (programmed death receptor-1 inhibitor), with or without GS-4774, a therapeutic T-cell vaccine, showed 3 (13.6%) patients in the high-dose arm achieved HBsAg reduction >0.5 log at week 24 and 1 patient (4.5%) had undetectable HBsAg lasting 12 months after therapy.^86^ A phase 2b study of envafolimab (ASC22), a humanized programmed cell death ligand-1 antibody in virally suppressed patients, showed 0.38 log decrease in HBsAg levels at 24 weeks with 3 patients (all had baseline HBsAg <100 IU/mL) achieving HBsAg loss. Mild ALT flares were observed in those with marked HBsAg decrease.^30^,^92^

#### Combination therapies

Combination therapies will be needed to achieve a functional cure. To date, only the following combinations have resulted in off-treatment HBsAg loss: NA+NAP+pegIFNα, ASO+/−NA, siRNA+peg-IFNα. Although the highest rate of functional cure was reported with NA+NAP+pegIFNα, these results need to be confirmed in larger trials, and the high frequency of marked ALT flares remains a concern. The results of bepirovirsen +/−NA showed the most promise though only one third of those who lost HBsAg at the end of treatment remained HBsAg negative 24 weeks later, and longer follow-up will be needed to determine the durability of HBsAg loss. Of note, although the addition of NA did not seem to make a difference in HBeAg-negative patients, a combination with NA seemed necessary in HBeAg-positive patients. Other combination therapies that have resulted in end-of-treatment HBsAg loss include PD-L1 antibody+NA or siRNA+neutralizing monoclonal antibody, whether these responses will be sustained off-treatment remain to be determined. Of note, adding CAM to siRNA+NA did not confer any benefit in 2 trials, whereas adding pegIFNα to siRNA seemed to increase the odds of HBsAg loss during treatment though the durability of response is unknown.

## STRATEGIES TOWARD HBV CURE

### Setting realistic goals

Functional HBV cure, defined as off-treatment sustained HBsAg loss, remains the goal of new HBV therapies. However, alternative endpoints may need to be adopted as intermediate steps to determine the success of clinical trials. The phase of clinical trials, the patient population studied, and the mode of action of the drugs tested will determine the choice of the most appropriate endpoints. At each AASLD-EASL HBV Treatment Endpoint Conference, representatives from the US Food and Drug Administration and the European Medicines Agency have expressed flexibility and encouraged industry sponsors to engage in consultative discussions when planning clinical trials. Achievement of any of the intermediate steps: decrease in HBsAg level to <100 IU/mL, complete silencing of cccDNA transcription, or partial cure after a finite course of treatment (eg, 2 years) in a high percentage of patients is a step forward compared with current therapies and are worthy goals in the foreseeable future.

### Having the right tools

**FIGURE 3:**
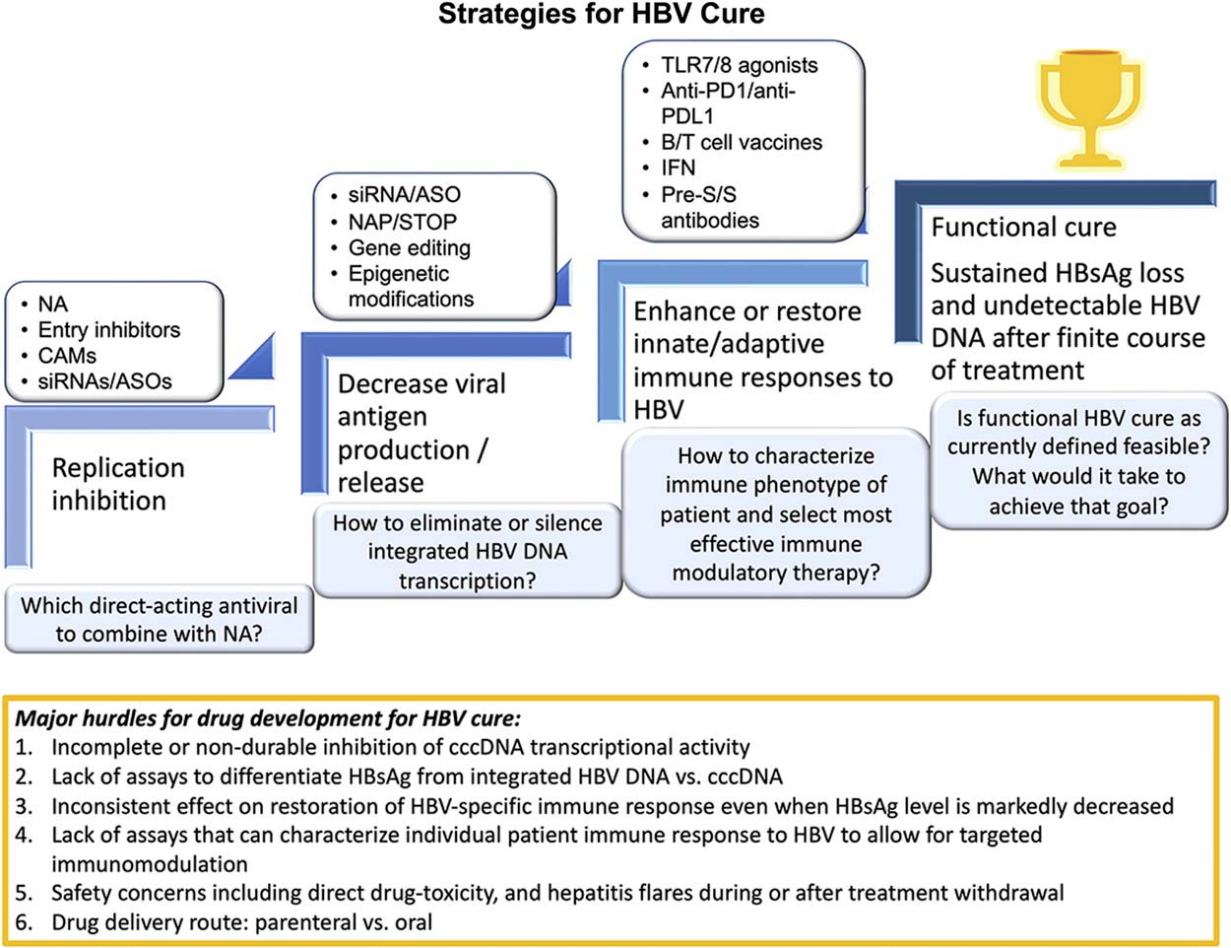
Strategies to achieve functional HBV cure and the current hurdles in developing novel agents against CHB. *De novo* or sequential combination of different classes of direct-acting antiviral drugs with or without immune modulatory therapies to inhibit HBV replication, decrease viral antigen production or release, and stimulate or restore immune response to HBV will be required to achieve functional HBV cure. NAs will remain the backbone for HBV replication inhibition. The addition of CAMs may provide more complete suppression of viral replication, the addition of entry inhibitors may prevent *de novo* infection of uninfected hepatocytes, and the addition of siRNA/ASO may further decrease viral replication. Translation inhibitors, siRNA, and ASO will be pivotal in decreasing viral protein production. PegIFNα can also decrease viral protein production in addition to its immunomodulatory effects. HBsAg release inhibitors, NAP and STOP, can decrease circulating HBsAg. Inhibition of HBV replication and viral protein production may be sufficient in restoring immune response to HBV in some patients while others will need immune modulatory therapies to stimulate/restore innate and/or adaptive immune response to achieve functional HBV cure. The availability of tests that can reliably determine when cccDNA transcription is completely silenced, the source of circulating HBsAg from cccDNA versus integrated HBV DNA, and patient’s immune response to HBV would be crucial to the development of HBV cure. The current major hurdles in drug development for CHB include incomplete or nondurable inhibition of cccDNA transcriptional activity, lack of effect on HBsAg production from integrated HBV DNA, and inconsistent effect on restoring HBV-specific immune response even when HBsAg level is markedly decreased. The knowledge gap and inadequate tools in assessing individual patient’s immune response to HBV hinder strategies to target immunomodulation. Safety is also a concern, as the development of several drugs was terminated because of drug-induced toxicity. Another safety concern relates to potential hepatitis flare during treatment and when treatment is discontinued. Drug delivery poses a concern for patient acceptance and global uptake, given that NAs are administered orally. New drugs that need to be given parenterally have to show significant incremental efficacy and comparable safety for patients to replace oral therapy with parenteral therapy. Drug resistance so far have been less of a concern because most trials extending for 12 or more weeks have been in combination with NA. Abbreviations: CAM, capsid assembly modulators; cccDNA, covalently closed circular DNA; NA, nucleos(t)ide analog; PD-1, programmed death receptor-1; siRNA, small interfering RNA.

Achievement of a functional HBV cure will require the complete suppression of HBV DNA replication, inhibition of HBsAg production, and restoration/boosting of innate and adaptive immune responses against HBV (Figure 3). It is envisioned that each step may require more than 1 class of drugs. Intuitively, the steps may need to occur in sequence, but the suppression of HBV DNA replication and HBsAg production may occur simultaneously, and some classes of antivirals, such as siRNA, ASO, and pegIFNα, can act at more than 1 step. In some patients, the complete suppression of HBV DNA replication and a marked decrease in HBsAg production may restore immune response with resultant HBsAg loss. However, many patients will require removal of immune blockade and/or boosting of immune responses to achieve sustained HBsAg loss.

NAs will remain a backbone in suppressing HBV DNA replication. CAMs, entry inhibitors, and translation inhibitors can also contribute.

Translation inhibitors, siRNA, and ASO will be pivotal in inhibiting HBsAg and other HBV protein production, allowing the recovery of the exhausted immune responses. PegIFNα can augment the inhibition of HBsAg production or facilitate HBsAg loss after HBsAg has been reduced to low levels. Other antivirals that may contribute to decreased HBsAg production include next-generation CAMs, entry inhibitors, and NAPs. Immune modulatory therapies such as therapeutic vaccines and checkpoint inhibitors can also contribute to inhibiting HBsAg production.

To achieve a high rate of HBsAg loss, HBsAg production from integrated HBV DNA needs to be inhibited by eliminating integrated HBV DNA through gene editing with a risk of off-target effects or generation of undesired mutations or eliminating hepatocytes that harbor integrated HBV DNA with risk of severe flares. Alternatively, silencing of integrated HBV DNA transcription may be achieved through epigenetic modifications, which may be more specific and reversible. A major challenge is differentiating whether HBsAg that remains in circulation is derived from cccDNA or integrated HBV DNA. This distinction is critical if complete suppression of cccDNA transcription is the endpoint of treatment. A recent study proposed that this can be achieved using a digital droplet PCR assay targeting midportion or 3′ terminal end of HBV S transcripts, the latter being absent in transcripts derived from integrated HBV DNA, but this assay relies on liver tissue and an assay that can be applied to blood samples is needed.^93^

The complete suppression of cccDNA transcription is best determined by quantifying intrahepatic pgRNA, but this is not practical for clinical trials. Surrogate serum markers, including HBV RNA and HBcrAg, which have been shown to correlate with cccDNA transcription, may be used if standardized assays with improved sensitivity for these markers are available.

Restoration of immune control is critical in sustaining HBsAg loss. Off-treatment reappearance of HBsAg in patients with undetectable HBsAg at the end of siRNA and ASO treatment trials has been attributed to lack of immune recovery. The availability of blood-based assays to characterize immune status would facilitate the identification of patients who need immunomodulatory therapies and the tailoring of these therapies to the immune phenotype of the patient. Although progress has been made, such assays/markers remain elusive.^94^

### Efficient design of clinical trials

Most trials have focused on patients with active disease virally suppressed on NA because they met guidelines criteria for treatment, constitute the majority of patients regularly followed in hepatology clinics, and have a low risk of hepatitis flares. However, this is a heterogeneous population with varying durations of NA therapy, a wide range in HBsAg levels and immune reconstitution, and a generally unknown HBV genotype. Post hoc analysis of several trials showed high rates of HBsAg loss in patients with low baseline HBsAg levels; future trials might enrich for such patients and possibly include inactive carriers, though it would be important to demonstrate whether the investigational therapies accelerated HBsAg loss in patients who are already on the path to HBsAg loss. Recent studies showed that the duration and not the phase of HBV infection belie the degree of immune exhaustion,^95^,^96^ and immunomodulatory therapies may be more likely to succeed in younger patients, including those in the immune tolerant phase. This hypothesis warrants testing. As inactive carriers and those in the immune tolerant phase have a very low risk of adverse clinical outcomes short term,^97^,^98^ these populations should only be enrolled in clinical trials of new drugs after safety in patients with active disease have been well established, for instance, in phase IIb or phase III clinical trials.

To date, most trials have used traditional designs testing one drug or one combination of drugs at a time. The efficiency can be improved using platform trials where multiple drugs can be tested using the same infrastructure, adding or removing new drugs based on initial results, and using the same controls for multiple comparisons. Platform trials can include patients with different characteristics channeled to the therapies that are most likely to succeed, enabling patients to be enrolled in the treatment arm that fit them best. Platform trials will require access to multiple drugs and collaborations among companies competing for the same goal.

Efficient enrollment is key to success of clinical trials. A major hurdle in enrolling patients in clinical trials of new HBV therapies is safety. NAs are administered orally and have excellent safety profile. Although many patients on long-term NA therapy desire to have their HBV infection cured, they are rightfully concerned about the risks of experimental therapies and inconvenience (many of the new drugs are parenterally administered, and clinical trials require frequent in-person assessments and testing) with uncertain benefit. Thus, stringent safety monitoring and detailed plans for managing adverse events, particularly hepatitis flares, and methods to differentiate potentially beneficial from harmful flares, must be in place. Although there is a strong desire for finite therapy, treatment withdrawal must be justified and closely monitored. Until recently, ALT is the focus of post-treatment safety monitoring and criteria for resuming treatment, but data from studies of NA withdrawal and new therapies have shown that a marked increase in HBV DNA levels often precede ALT flares and might be a safer trigger for reinitiating treatment.^36^,^99^

## CONCLUDING PERSPECTIVES

Sustained HBsAg loss can be accomplished in selected patients with current therapies, for example, pegIFNα in HBeAg-positive patients with genotype A, and NA withdrawal in White HBeAg-negative patients with HBsAg level <1000 IU/mL. HBsAg loss has also been observed in the clinical trials of some new therapies, for example, checkpoint inhibitor and NA, ASO with or without NA, NAP with NA and pegIFNα, predominantly in patients with low baseline HBsAg levels. Expanding the options for these “easy-to-cure” patients should be encouraged, but effort to find a cure for the majority left behind must continue. Clinical trials of drugs that have shown efficacy in decreasing cccDNA transcription or HBsAg production should continue, and the achievement of intermediate endpoints should propel further development, which may include collaboration among pharmaceutical companies and the design of platform trials comparing multiple drug combinations with the matching of patients to the best-suited treatment.

Although antiviral approaches alone may result in sustained HBsAg loss in some patients, a functional HBV cure will likely require immunomodulatory therapies in many patients. The development of reliable immunologic assays or markers to identify which patients need immunomodulatory therapies and how to tailor the choice of immunomodulatory therapy to maximize the likelihood of HBsAg loss is urgently needed. Similarly, the availability of reliable blood-based assays that can determine the source of circulating HBsAg is needed to select therapies directed at the right target and to assess response.

Although the previous goal of 30% sustained HBsAg loss after 1–2 years of treatment might seem more remote now than in 2016, the efforts invested in the last 5 years have provided important tools and insights toward achieving that goal. Stakeholders, including regulatory agencies, pharmaceutical industry, scientists, clinicians, and patients, should promote collaborations and conduct of platform trials with treatment arms tailored according to patient characteristics to safely achieve functional HBV cure in incremental steps.

## Supplementary Material
