# Supplementary material for: What will it take to cure hepatitis B?
Source: Hepatol Commun. 2023 Mar 24;7(4):e0084. doi: 10.1097/HC9.0000000000000084 (PMC10043561; doi:10.1097/HC9.0000000000000084)
Supplement: Supplementary file 1 [file hc9-7-e0084-s001.docx]

**Supplementary: References for Table 2 (Listed from the latest date)**

1. **Translation inhibitors**
   1. **siRNA**

- ***JNJ-3989 (JNJ-73763989)***

1. Agarwal K, Buti M, Van Bommel F, Lampertico P, Janczewska E, Bourliere M, et al. Efficacy and safety of combination treatment with siRNA JNJ-73763989 and capsid assembly modulator JNJ-56136379 (Bersacapavir) in HBeAg negative virologically suppressed chronic hepatitis B (CHB) patients: follow-up week 48 end of study results from REEF-2. Hepatology. 2022;76(S1):LB5012
2. Gane EJ, Janczewska E, Takehara T, Chuang WL, Peng CY, Hlebowicz M, et al. Efficacy and safety of siRNA JNJ-73763989, capsid assembly modulator JNJ-56136379, nucleos(t)ide analog (NA), and pegylated interferon alpha-2a (PEGIFNα2a) for treatment of chronic hepatitis B (CHB): week 24 results from the phase 2 PENGUIN study. Hepatology. 2022;76(S1):LB5035
3. Yuen MF, Locarnini S, Lim TH, Strasser SI, Sievert W, Cheng W, et al. Combination treatments including the small-interfering RNA JNJ-3989 induce rapid and sometimes prolonged viral responses in patients with CHB. J Hepatol. 2022 Nov;77(5):1287-1298
4. Agarwal K, Buti M, Van Bommel F, Lampertico P, Janczewska E, Bourliere M, et al. Efficacy and safety of finite 48-week treatment with the siRNA JNJ-3989 and the capsid assembly modulator (CAM-N) JNJ-6379 in HBeAg negative virologically suppressed (VS) chronic hepatitis B (CHB) patients: results from REEF-2 study. J Hepatol. 2022;77:S8
5. Yuen MF, Asselah T, Jacobson IM, Brunetto M, Janssen H, Takehara T, et al. Effects of the siRNA JNJ-3989 and/or the capsid assembly modulator (CAM-N) JNJ-6379 on viral markers of chronic hepatitis B (CHB): results from the REEF-1 study. J Hepatol. 2022;77:S864-S865.

- ***VIR-2218***

1. Gane E, Jucov A, Dobryanska M, Yoon KT, Lim TH, Arizpe A, et al. Safety, tolerability and antiviral activity of the siRNA VIR-2218 in combination with the investigational neutralizing monoclonal antibody VIR-3434 for the treatment of chronic hepatitis B infection: Preliminary results from the phase 2 MARCH trial. Hepatology. 2022;76(S1):S18
2. Yuen MF, Lim YS, Cloutier D, Thanawala V, Shen L, Elie SE, et al. Preliminary 48-week safety and efficacy data of VIR-2218 alone and in combination with pegylated interferon alfa in participants with chronic HBV infection. Hepatology. 2022;76(S1):S19
3. Lim YS, Yuen MF, Cloutier D, Thanawala V, Shen L, Gupta SV, et al. Longer treatment duration of monthly VIR-2218 results in deeper and more sustained reductions in hepatitis B surface antigen in participants with chronic hepatitis B infection. J Hepatol. 2022;77:S69-S70.
4. Gane E, Lim YS, Cloutier D, Shen L, Cathcart A, Ding X, et al. Safety and antiviral activity of VIR-2218, an X-targeting RNAi therapeutic, in participants with chronic hepatitis B infection: week 48 follow-up results. J Hepatol. 2021;75:S287-S288.

- ***AB-729***

1. George J, Stefanova-Petrova D, Antonov K, Valaydon Z, Davison S, Fung S, et al. Evaluation of the Vebicorvir, NRTI and AB-729 combination in virologically suppressed patients with HBeAg negative chronic hepatitis B virus infection: interim analysis from an open label phase 2 study. Hepatology. 2022;76(S1):LB5064.
2. Yuen MF, Holmes J, Strasser SI, Leerapun A, Sukeepaisarnjaroen W, Tangkijvanich P, et al. Hepatitis B viral control maintained during extended follow up of HBeAg- chronic hepatitis B (CHB) subjects who discontinued nucleos(t)ide analogue (NA) therapy after completion of AB-729 treatment, and in HBeAg+ subjects still on NA therapy Hepatology. 2022;76(S1):LB5047.
3. Ganchua SC, Paratala B, Iott C, Gane EJ, Yuen MF, Eley T, et al. Reduction of hepatitis B surface antigen mediated by RNA interference therapeutic AB-729 in chronic hepatitis B patients is associated with T cell activation and a decline in exhausted CD8 T cells. J Hepatol. 2022;77:S851.
4. Yuen MF, Berliba E, Sukeepaisarnjaroen W, Holmes J, Leerapun A, Tangkijvanich P, et al. Long-term suppression maintained after cessation of AB-729 treatment and comparable on-treatment response observed in HBeAg positive subjects. J Hepatol. 2022;77:S876-S877.
5. Yuen MF, Strasser S, Sukeepaisarnjaroen W, Sharma V, Antoniello D, Medvedeva E, et al. Continued suppression of viral markers observed following discontinuation of nucleos (t)ide analogue therapy in chronic hepatitis B subjects with low hepatitis B surface antigen levels after 48 weeks of treatment with AB-729. J Hepatol. 2022;77:S878-S879.

- 1. **ASO**
- **Bepirovirsen (GSK3228836) (B-Clear)**

1. Yuen MF, Lim SG, Plesniak R, Tsuji K, Janssen HLA, Pojoga C, et al. Efficacy and Safety of Bepirovirsen in Chronic Hepatitis B Infection. N Engl J Med. 2022. Epub 20221108. doi: 10.1056/NEJMoa2210027.
2. You S, Delahaye J, Ermler M, Singh J, Jordan W, Ray A, et al. Bepirovirsen, antisense oligonucleotide (ASO) against hepatitis B virus (HBV), harbors intrinsic immunostimulatory activity via Toll-like receptor 8 (TLR8) preclinically, correlating with clinical efficacy from the Phase 2a study. J Hepatol. 2022;77:S873-S874.
3. Yuen MF, Heo J, Kumada H, Suzuki F, Suzuki Y, Xie Q, et al. Phase IIa, randomised, double-blind study of GSK3389404 in patients with chronic hepatitis B on stable nucleos(t)ide therapy. J Hepatol. 2022 Oct;77(4):967-977.
4. Yuen MF, Heo J, Jang JW, Yoon JH, Kweon YO, Park SJ, et al. Safety, tolerability and antiviral activity of the antisense oligonucleotide bepirovirsen in patients with chronic hepatitis B: a phase 2 randomized controlled trial. Nat Med. 2021;27(10):1725-34.

1. **Capsid assembly modulators**

- **ABI-H0731**

1. Fung S, Sulkowski M, Lalezari J, Schiff ER, Dieterich D, Hassanein T, et al. Antiviral activity and safety of the hepatitis B core inhibitor ABI-H0731 administered with a nucleos(t)ide reverse transcriptase inhibitor in patients with HBeAg-negative chronic hepatitis B infection. J Hepatol. 2020;73:S51-S52.
2. Yuen MF, Agarwal K, Ma XL, Nguyen T, Schiff ER, Hann HW, et al. Antiviral activity and safety of the hepatitis B core inhibitor ABI-H0731 administered with a nucleos(t)ide reverse transcriptase inhibitor in patients with HBeAg-positive chronic hepatitis B infection in a long-term extension study. J Hepatol. 2020;73:S140

- **RG7907 (RO7049389)**

1. Hou JL, Gane EJ, Zhang WH, Zhang JM, Yuen MF, Lim TH, et al. Hepatitis B virus antigen reduction effect of RO7049389 plus NUC with/without Peg-IFN in chronic hepatitis B patients. J Hepatol. 2022;77:S299
2. Yuen MF, Balabanska R, Hou JL, Gane EJ, Lim TH, Zhang WH, et al. Viral nucleic acids suppression activity of RO7049389 plus NUC with/without Peg-IFN in virologically-suppressed and naive chronic hepatitis B patients: 48-week treatment and post-treatment follow-up. J Hepatol. 2022;77:S867

- **JNJ-6379 (JNJ-56136379)**

1. Agarwal K, Buti M, Van Bommel F, Lampertico P, Janczewska E, Bourliere M, et al. Efficacy and safety of combination treatment with siRNA JNJ-73763989 and capsid assembly modulator JNJ-56136379 (Bersacapavir) in HBeAg negative virologically suppressed chronic hepatitis B (CHB) patients: follow-up week 48 end of study results from REEF-2. Hepatology. 2022;76(S1):LB5012
2. Gane EJ, Janczewska E, Takehara T, Chuang WL, Peng CY, Hlebowicz M, et al. Efficacy and safety of siRNA JNJ-73763989, capsid assembly modulator JNJ-56136379, nucleos(t)ide analog (NA), and pegulated interferon alpha-2a (PEGIFNα2a) for treatment of chronic hepatitis B (CHB): week 24 results from the phase 2 PENGUIN study. Hepatology. 2022;76(S1):LB5035
3. Agarwal K, Buti M, Van Bommel F, Lampertico P, Janczewska E, Bourliere M, et al. Efficacy and safety of finite 48-week treatment with the siRNA JNJ-3989 and the capsid assembly modulator (CAM-N) JNJ-6379 in HBeAg negative virologically suppressed (VS) chronic hepatitis B (CHB) patients: results from REEF-2 study. J Hepatol. 2022;77:S8
4. Yuen MF, Locarnini S, Lim TH, Strasser SI, Sievert W, Cheng W, et al. Combination treatments including the small-interfering RNA JNJ-3989 induce rapid and sometimes prolonged viral responses in patients with CHB. J Hepatol. 2022. Epub 20220720. doi: 10.1016/j.jhep.2022.07.010.
5. Yuen MF, Asselah T, Jacobson IM, Brunetto M, Janssen H, Takehara T, et al. Effects of the siRNA JNJ-3989 and/or the capsid assembly modulator (CAM-N) JNJ-6379 on viral markers of chronic hepatitis B (CHB): results from the REEF-1 study. J Hepatol. 2022;77:S864-S865.
6. Janssen H, Hou J, Asselah T, Chan H, Zoulim F, Tanaka Y, et al. Efficacy and safety results of the phase 2 JNJ-56136379 JADE study in patients with chronic hepatitis B: Interim week 24 data. J Hepatol. 2020;73:S129-S130.

- **GLS-4**

1. Zhang H, Wang F, Zhu X, Chen Y, Chen H, Li X, et al. Antiviral Activity and Pharmacokinetics of the Hepatitis B Virus (HBV) Capsid Assembly Modulator GLS4 in Patients With Chronic HBV Infection. Clin Infect Dis. 2021;73(2):175-182.

1. **NAP**

- ***REP 2139 and REP 2165***

1. Bazinet M, Pantea V, Placinta G, Moscalu I, Cebotarescu V, Cojuhari L, et al. Safety and Efficacy of 48 Weeks REP 2139 or REP 2165, Tenofovir Disoproxil, and Pegylated Interferon Alfa-2a in Patients With Chronic HBV Infection Naive to Nucleos(t)ide Therapy. Gastroenterology. 2020;158(8):2180-2194.
2. **Remove immune blockade**

**PDL1 inhibitor**

- ***Envafolimab(ASC22)***

1. Wang GQ, Cui YM, Xie Y, Mao QG, Xie Q, Gu Y, et al. ALT flares were linked to HBsAg reduction, seroclearance and seroconversion: interim results from a phase IIb study in chronic hepatitis B patients with 24-week treatment of subcutaneous PD-L1 Ab ASC22 (Envafolimab) plus nucleos (t)ide analogs. J Hepatol. 2022;77:S70
2. Wang GQ, Cui YM, Xie Y, Mao QG, Xie Q, Ye G, et al. HBsAg loss in chronic hepatitis B patients with subcutaneous PD-L1 antibody ASC22 (Envafolimab) plus nucelos(t)ide analogs treatment: interim results from a phase IIb clinical trial. HEPATOLOGY. 2021;74(6):1392A
3. **Therapeutic vaccine**

- ***GS-4774***

1. Boni C, Janssen HLA, Rossi M, Yoon SK, Vecchi A, Barili V, et al. Combined GS-4774 and Tenofovir Therapy Can Improve HBV-Specific T-Cell Responses in Patients With Chronic Hepatitis. Gastroenterology. 2019;157(1):227-241.
2. Gane E, Verdon DJ, Brooks AE, Gaggar A, Nguyen AH, Subramanian GM, et al. Anti-PD-1 blockade with nivolumab with and without therapeutic vaccination for virally suppressed chronic hepatitis B: A pilot study. J Hepatol. 2019;71(5):900-907.
3. Janssen HL, Yoon SK, Yoshida EM, Trinh HN, Rodell TC, Nguyen AH, et al. Safety and Efficacy of GS-4774 in combination with TDF in Patients with Chronic Hepatitis B not on Antiviral Medication. HEPATOLOGY. 2016;64:122A
4. Lok AS, Pan CQ, Han SHB, Trinh HN, Fessel WJ, Rodell T, et al. Randomized phase II study of GS-4774 as a therapeutic vaccine in virally suppressed patients with chronic hepatitis B. J Hepatol. 2016;65(3):509-516.

- ***VBI-2601***

1. Ma H, Lim TH, Leerapun A, Weltman M, Jia J, Lim YS, et al. Therapeutic vaccine BRII-179 restores HBV-specific immune responses in patients with chronic HBV in a phase Ib/IIa study. JHEP Rep. 2021;3(6):100361.

- ***VTP-300***

1. Evans T, Barnes E. Phase 1b/2a study of heterologous ChAdOx1-HBV/MVA-HBV therapeutic vaccination (VTP-300) combined with low-dose nivolumab (LDN) in virally-suppressed patients with CHB on nucleos (t)ide analogues. J Hepatol. 2022;77:S868
2. **Restore innate immunity**

**TLR 7 agonist**

- ***GS-9620 (vesatolimod)***

1. Agarwal K, Ahn SH, Elkhashab M, Lau AH, Gaggar A, Bulusu A, et al. Safety and efficacy of vesatolimod (GS-9620) in patients with chronic hepatitis B who are not currently on antiviral treatment. J Viral Hepat. 2018;25(11):1331-1340.
2. Boni C, Vecchi A, Rossi M, Laccabue D, Giuberti T, Alfieri A, et al. TLR7 Agonist Increases Responses of Hepatitis B Virus-Specific T Cells and Natural Killer Cells in Patients With Chronic Hepatitis B Treated With Nucleos(T)Ide Analogues. Gastroenterology. 2018;154(6):1764-1777.
3. Janssen HLA, Brunetto MR, Kim YJ, Ferrari C, Massetto B, Nguyen AH, et al. Safety, efficacy and pharmacodynamics of vesatolimod (GS-9620) in virally suppressed patients with chronic hepatitis B. J Hepatol. 2018;68(3):431-440.

**TLR 8 agonist**

- ***GS-9688 (selgantolimod)***

1. Janssen HLA, Lim YS, Kim HJ, Tseng CH, Coffin CS, Elkashab M, et al. Safety and efficacy of oral TLR8 agonist, selgantolimod, in viremic adult patients with chronic hepatitis B. J Hepatol. 2021;75:S757-S758.
2. Gane E, Dunbar PR, Brooks A, Zhao Y, Tan S, Lau A, et al. Efficacy and safety of 24 weeks treatment with oral TLR8 agonist, selgantolimod, in virally-suppressed adult patients with chronic hepatitis B: a phase 2 study. J Hepatol. 2020;73:S52-S.
3. Khanam A, Yoon JA, Poonia B, Grant E, Kottilil S, Tang L. GS-9688, a Toll-like Receptor 8 Agonist Induces Innate and Adaptive Antiviral Immune Response in Chronic Hepatitis B Patients. HEPATOLOGY. 2018;68:329A-330A.
